# Acknowledgment to Reviewers of *Medicines* in 2020

**DOI:** 10.3390/medicines8020007

**Published:** 2021-01-20

**Authors:** Medicines Editorial Office

**Affiliations:** MDPI AG, St. Alban-Anlage 66, 4052 Basel, Switzerland; medicines@mdpi.com

Peer review is the driving force of journal development, and reviewers are gatekeepers who ensure that *Medicines* maintains its standards for the high quality of its published papers. Thanks to the cooperation of our reviewers, in 2020, the median time to first decision was 18 days and the median time to publication was 36 days. The editors would like to express their sincere gratitude to the following reviewers for their precious time and dedication, regardless of whether the papers were finally published:
Adhikari-Devkota, AnjanaMorais, NunoAghanavesi, SomayehMoran-Lev, HadarAlconchel, FelipeMorla, ShravanAsakawa, TetsuyaMorouço, PedroBaj, JacekMurillo-Saich, Jessica D.Banerjee, SudipNagahama, YasunoriBang, SangsuNedialkov, Paraskev T.Barfod, Kristoffer WeisskirchnerNekludov, MichaelBaumgard, LanceNiakšu, OlegasBaumgarden, JosephNitulescu, MihaiBeaune, SébastienOhnishi, KohtaBelluzzi, ElisaOo, Win MinBenarba, BachirOrtega, Angel L.Benzeroual, Kenza E.Osborne, NickBontempo, PaolaOwczarek, AleksandraBorel-Hänni, FrançoisPaine, AnantaBose, TanimaPalazzuoli, AlbertoBraga, Susana SantosPallag, AnnamáriaBREZOIU, Ana-MariaParajuli, NirmalaBruzzese, DarioPark, HangueBujak, TomaszParodi, Pier CamilloCaggiati, AlbertoPawełczyk, AnnaCalina, DanielaPayne, PeterCapasso, RaffaelePedraza Chaverri, JoséCatani, MartinaPellesi, LanfrancoCatela, DavidPilmane, MāraCelakovska, JarmilaPiriou, YannickCervera-Garvi, PabloPlant, LeighCheng, Hung-ChiPolańczyk, AndrzejCheungpasitporn, WisitPolewczyk, AnnaCho, Jang-HeePop, Gheorghe NicusorChung, EricPrimorac, DraganÇiçek, Serhat SezaiPrior, SarahClanton, ThomasProdromos, Chadwick C.Coldea, Teodora EmiliaProtheroe, AndrewCosma, Maria PiaPsurski, MateuszCostantini, ElisabettaPyka-Pająk, AlinaCriado, BegoñaR. El-Seedi, HeshamDamsky, William E.Raheem, Omer A.D’Andrea, SettimioRavin, James G.De Francesco, FrancescoReid, Terry-ElinorDel Rio, AlessandroRevuelta-Herrero, José LuisDemirhan, ErhanRogobete, Alexandru FlorinDi Somma, SalvatoreRogozea, LilianaDiogo, PatriciaRóżalska, BarbaraEhrentraut, Stefan FelixRubio-Arraez, S.Eibenschutz, LauraRungratanawanich, WiramonEisenstat, David D.Sadan, OferErb, CarlSaito, AyaFalhammar, HenrikSaito, YukihiroFernandez-Campos, FranciscoSakakibara, HiroyukiFigueira, Maria EduardoSalati, MassimilianoFleitas, TaniaSaldana, Santiago J.Frabboni, LauraSallée, MarionFujita, MasakiSam, RaminGabryelska, AgataSanda, MiloslavGalache Osuna, CristinaSantos-Costa, QuirinaGalassi, AndreaSasso, Ferdinando CarloGhosh, ArnabSaul, DominikGłąbska, DominikaSchirmer, BastianGobbi, AlbertoSciortino, Maria TeresaGorgey, Ashraf S.Sethi, GautamHallgren, MatsShakya, AkhileshHare, Joshua M.Shibata, YasushiHayashi, YoheiSinger, Bernhard B.Ho, Cheng-MawSorrell, J. MichaelHolsinger, DamainStaerman, FredericIbáñez-Vera, Alfonso JavierStankovic, MilanIerardi, EnzoStano, PasqualeJanczar, SzymonStefani, LauraJoshi, SameerSterodimas, ArisJung, Ernst-MichaelStudzinska-Sroka, ElzbietaJurjus, Rosalyn A.Subramani Paranthaman, BalasubramaniKairuz, ThereseSugimoto, MitsushigeKamimura, KenyaSuzuki, RyuichiroKarbownik-Lewińska, MałgorzataTaguchi, ToruKawauchi, HideyukiTedesco, IdoloKhairullina, VeronikaTeixeira, LeandroKlimczak, AleksandraTennant, Matthew T.s.Klontzas, MichailTeragawa, HirokiKong Thoo Lin, PaulThompson, Miles D.Krijt, JanTinoco, Arthur D.Larese, FrancescaTrivanovic, DrenkaLee, Yu-ChenTsagareli, Merab G.Lehmann, ChristianTurrini, EleonoraLevkovich, InbarTwohig, HelenLevy, StevenUrzua, AlejandroLewinska, AnnaValentová, KateřinaLewis, JamesValero-Ramón, ZoeLewitus, DanVelez, ZéliaLin, Chien-MinVetter, Stefan W.Lipshultz, Larry I.Vila-Suárez, Maria ElenaLitofsky, Norman ScottVillena, JuanLiu, Cheuk LunWang, David Q.Liu, WenWang, XinyuLiu, XiaoxiWechalekar, Mihir DilipLiu, Yen BinWeiss, Jeffrey N.Locatelli, GiuseppeWojtkiewicz, JoannaLu, PaulWollebo, Hassen S.Ma, XiaochaoWu, (Jason) BoyangMangalagiu, IonelYilmaz, BahtiyarMangone, LuciaYoshioka, Ken-ichiMarbacher, SergeYu, Chia-LiMartina, LambertiniYue, John K.Marx, Joannes J. M.Yunusa, IsmaeelMayer, OttoZakrocka, IzabelaMaynar Mariño, MarcosZhang, ChiMitchell, Cassie S.Zimova, LucieMlynarski, RafalZingaretti, NicolaMolina-Leyva, Alejandro


